# New approach for the treatment of osteoradionecrosis with pentoxifylline and tocopherol

**DOI:** 10.1186/2055-7124-18-13

**Published:** 2014-09-29

**Authors:** Huan Fan, Soung Min Kim, Yun Ju Cho, Mi Young Eo, Suk Keun Lee, Kyung Mi Woo

**Affiliations:** Department of Oral and Maxillofacial Surgery, Dental Research Institute, School of Dentistry, Seoul National University, 62-1 Changgyeonggungno, Jongno-gu, Seoul, 110-768 South Korea; Department of Oral Pathology, College of Dentistry, Gangneung-Wonju National University, Gangneung, South Korea; Department of Dental Pharmacology & Therapeutics, Dental Research Institute, School of Dentistry, Seoul National University, Seoul, South Korea

**Keywords:** Osteoradionecrosis (ORN), Pentoxifylline, Tocopherol, Radiotherapy

## Abstract

Osteoradionecrosis (ORN) of the jaw is a significant complication of radiotherapy for oral cavity cancer. In addition to antibiotic medication, treatment options such as hyperbaric oxygen therapy, surgical approaches, and combined therapy with pentoxifylline and tocopherol have been recently introduced.

In this review article, we will discuss the definition and classifications of osteoradionecrosis, its etiology and pathophysiology, previous treatment options, oral and maxillofacial complications of radiotherapy, basic information on pentoxifylline and tocopherol, recent reports of pentoxifylline and tocopherol combined therapy, and, finally, ORN-induced animal models and future approaches.

## Introduction

**Figure 1:**
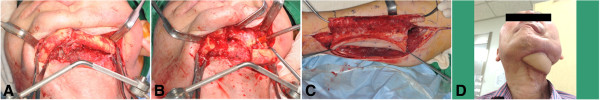
**Clinical views of surgical approaches in an ORN patient, including the identification and resection of the necrotic mandible with associated soft tissues (A, B), the harvesting of the composite fibular free flap from the left lower leg of the patient (C), and a two-week post-operative view of the patient (D).**

Osteoradionecrosis (ORN) of the jaw is one of the main complications of radiotherapy, and results in alteration of the shape and function of the oral cavity and jaw that causes substantial deterioration in patient quality of life (Figure 1). Although the incidence of ORN has declined recently with the advancement in radiation techniques and increased focus on the predisposing factors for ORN, the pathogenesis and pathophysiology of ORN remain unclear, and its risk factors have not completely been elucidated.

Pentoxifylline has been marketed in Europe for the management of vascular disorders such as intermittent claudication, and has been found to act against some inflammatory mediators including TNF-α. Alpha-tocopherol, as named vitamin E, scavenges free radicals generated during oxidative stress and protects cell membranes against lipid peroxidation [1, 2]. Given these well-known antioxidant properties of tocopherol, these two drugs have recently been reported to be potent and synergistic antifibrotic agents.

During our investigations of the pathophysiology of ORN in a radiation-induced animal model with a tissue engineering approach [3], we noticed the efficacy of combined pentoxifylline and tocopherol therapy. To advance the knowledge of biomaterial applications in the treatment of ORN, this article is intended to provide a review of the current trends for conservative treatment in ORN of the jaw.

## Review

### Osteoradionecrosis

**Figure 2:**
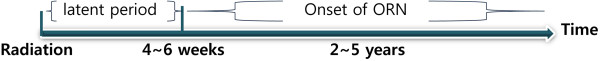
**Schematic timetables of the duration of the latent period, and the onset of osteoradionecrosis.**

Based on clinical findings, ORN of the jaw can be defined as irradiated bone that becomes devitalized and exposed through overlying skin or mucosa without healing for three months, without recurrence of cancer. Two criteria in the definition, the duration of exposure and the meaning of devitalization, have been controversial. ORN has been defined as “when bone in the radiation field is exposed for at least 2 months in the absence of local neoplastic disease”, “an area greater than 1 cm of exposed bone in a field of irradiation that fails to show any evidence of healing for at least 6 months” [4, 5], “an area of exposed mandible present for longer than 2 months in a previously irradiated field, in the absence of recurrent tumor” [5, 6], “an ulceration of the mucous membrane with exposure of necrotic bone”, and “irradiated bone [that] becomes devitalized and exposed through the overlying skin or mucosa, persisting without healing for 3 months in the absence of tumor recurrence” [1, 7]. Generally, a duration of four to six weeks can be accepted (Figure 2), and devitalization can be defined as radiologic findings of bony necrosis through histologic confirmation of necrotic bone within the radiation field [8, 9]. ORN has also been described as radiation osteitis, radio-osteonecrosis, radiation osteomyelitis, osteomyelitis of irradiated bone, osteonecrosis, radio-osteomyelitis, septic osteoradionecrosis, and post-radiotherapy osteonecrosis [1].

The incidence of ORN after radiotherapy for head and neck cancers has been reported to be due to the loss of soft tissue, which naturally recovers, and the exposure of necrotic bone for over 6 months. The prevalence rate also varies widely, from less than 1% to as high as 30%, with a range of 10 to 15% reported in most literature. ORN affects the mandible more often than the maxilla, with an incidence between 2% and 22% [1, 10]. The disorder is rare after radiation of less than 60 Gy, but more common when brachytherapy is used, and less common after hyperfractionated radiotherapy at 72 to 80 Gy, or after moderately accelerated fractionated radiotherapy with a boost of 64 to 72 Gy [1, 11]. The incidence of ORN may be higher for concurrent chemotherapy and radiotherapy (CCRT), whereas it may be lower for intensity-modulated radiotherapy (IMRT) [1, 12–14]. The basic characteristics of ORN are summarized in Table 1.

**Table 1:** **Characteristics, risk factors, conservative and surgical treatment of ORN [** [9–13]

	Osteoradionecrosis of jaw
Characteristics	Irradiated bone becomes devitalized and exposed through the overlying skin or mucosa without healing for 3months, without recurrence of tumor
	Most case happen in mandible
	70-94% of cases developed within the first 3 years after radiotherapy
Risk factors	Hyperfractionated irradiation regimen - High total dose (6000-7000cGy)
	Recent reports have suggested that when chemotherapy is added to radiotherapy the incidence of ORN may be increased
	Pre-irradiation and post-irradiation dental extractions
	Poor oral hygien with periodontal disease
	Tobacco and alcohol use
Conservative treatment	Improve oral hygiene
	Minimal surgical debridement
	Hyperbaric oxygen therapy (HBOT)
	Medical management: pentoxifylline, tocopherol
Surgical treatment	Sequestrectomy, Saucerization
	Segmental resection and Free flap reconstruction

There are many different staging systems for ORN that have been published for clinical treatment and research (Tables 2, 3 and 4) [4, 8, 15]. These classifications were based on various criteria, such as soft tissue dehiscence, necrotic bone, oro-cutaneous fistula and pathologic fracture. Marx’s staging system is perhaps the most widely used and is predicated on staging ORN based on response to treatment [4].

**Table 2:** **Classification of ORN by Marx [** [1]

Stage	Description
I	Exposed alveolar bone without pathologic fracture, which responds to hyperbaric oxygen therapy
II	Disease does not respond to HBOT, and requires sequestrectomy and saucerization
III	Full thickness bone damage or pathologic fracture, usually requires complete resection and reconstruction with free tissue

**Table 3:** **Classification of ORN by Epstein et al. [** [7]

Stage	Description	Symtoms	Treatment
I	Resolved, healed	None	Follow-up, prevention of recurrence
Ia	No pathologic fracture		
Ib	Pathologic fracture		Reconstructed
II	Chronic, persistent non-progressive	None, or controlled	Local wound care
			Antiseptics/antibiotics, analgesics, hyperbaric oxygen (if indicated)
IIa	No pathologic fracture		
IIb	Pathologic fracture	Jaw dysfunction	
III	Active, preogressive	Progressive	Local wound care
			Antiseptics/antibiotics, analgesics, hyperbaric oxygen (if indicated)
IIIa	No pathologic fracture		
IIIb	Pathologic fracture	Jaw dysfunction

**Table 4:** **Classification of ORN by Notani et al. [** [14]

Grade	Description
I	ORN confined to alveolar bone
II	ORN limited to the alveolar bone and/or mandible aboce the level of the inferior alveolar canal
III	ORN involving the mandible below the level of the inferior alveolar canal and/or skin fistula and/or pathological fracture

The diagnosis of ORN is based primarily on clinical signs and symptoms. Ulceration or necrosis of the mucous membrane with exposure of necrotic bone is frequent and accompanied by neurologic symptoms of pain, dysesthesia or anesthesia, which may be present in the distribution of the inferior alveolar nerve in the mandible. Associated symptoms of fetor oris, dysgeusia, and food impaction may also be found. Exposure of rough, irregular denuded bone may result in physical irritation to the adjacent tissues, and progression of these conditions may lead to pathologic fracture and intra- or extraoral fistula formations. Functional disturbances with limitations in mouth opening and difficulties in mastication, deglutition and speech may also occur [8].

### Etiology and pathophysiology of osteoradionecrosis

Regaud published the first report on ORN of the jaw after radiotherapy in 1922, and Ewing reported in 1926 on the bone changes associated with radiation therapy and described this disease state as “radiation osteitis” [1, 16]. In 1938, Watson and Scarborough described this radiation osteitis as caused by radiation, trauma and infection. Trauma to the soft tissues overlying bone in the oral cavity induced bacteria to enter into the underlying demineralized bone and lead to osteomyelitis [1, 17]. Meyer classified ORN as a special type of osteomyelitis in 1970 [1, 18]. In 1972, Daly focused on the role of trauma in ORN and on the surface contaminant role of microorganisms, which is not the true etiological cause of ORN. In 1983, Marx redefined the pathophysiology of ORN by proposing that radiation therapy induces an endarteritis that results in tissue hypoxia, hypocellularity, and hypovascularity, which in turn causes tissue breakdown and chronic non-healing wounds [4]. In 1990, Bras et al. suggested from their histopathologic findings on specimens after sequestrectomy and resection that radiation-induced obliteration of the inferior alveolar artery was the dominant factor leading to ischemic necrosis of the mandible. Harris introduced the use of ultrasound as a modality to treat ORN in 1992 [7].

For the critical appraisal of the pathophysiology of ORN, associated risk factors of ORN can be investigated, including the primary site of cancer (more commonly the posterior mandible due to its dense bony nature), proximity of tumor to bone, extent of the mandible included in the primary radiation field, poor oral hygiene including odontogenic and periodontal disease, state of dentition, radiation dose above 60 Gy, nutritional status, concomitant chemo-radiation, chronic trauma from ill-fitting prostheses, and acute trauma from surgical procedures of the jaw.

There are some theories proposing that ORN is not a primary infection of irradiated bone, but rather a complex metabolic and tissue homeostatic deficiency created by radiation-induced cellular injury. Meyer proposed a theory involving radiation, trauma and infection and reported that oral microbiological flora invade the underlying irradiated bone after injury [1]. Endothelium, bone, and periosteum are all important tissues that have been shown to become hypoxic, hypocellular and hypovascular as a result of ORN [19]. With this theory, the classic sequence of radiation, trauma and infection can be replaced by a sequence of metabolic and cellular changes as cellular death and collagen lysis exceed synthesis and cellular replication, resulting in chronic non-healing wounds [4].

Recently, a new theory known as the “fibro-atrophic theory” has emerged, and proposes that fibroblast populations not only undergo total cellular depletion in response to radiation exposure, but also show a reduced ability to produce and secrete collagen into the surrounding tissue. This theory is based on the concept that osteoclasts suffer radiation damage earlier than the development of vascular alterations [1, 20]. Accordingly, the key event in the progression of ORN is the activation and dysregulation of fibroblastic activity that leads to atrophic tissue within a previously irradiated area. [1] The histopathologic phases of the development of ORN include the prefibrotic phase, the constitutive organized phase, and the late fibroatrophic phase. In the initial prefibrotic phase, changes in endothelial cells predominate, along with the acute inflammatory response; in the constitutive organized phase, abnormal fibroblastic activity predominates, and there is disorganization of the extracellular matrix; in the late fibroatrophic phase, tissue remodeling occurs along with the formation of fragile healed tissues that carry a serious inherent risk of late reactivated inflammation in the event of local injury [20, 21].

After radiotherapy, the endothelial cells are injured both from direct radiation damage and from indirect damage by radiation-generated reactive oxygen species or free radicals. Injured endothelial cells produce chemotactic cytokines that trigger an acute inflammatory response and then generate a further release of reactive oxygen species from polymorphs and other phagocytes. The destruction of endothelial cells, coupled with vascular thrombosis, lead to necrosis of microvessels, local ischemia, and tissue loss. Loss of the natural endothelial cell barrier results in the excretion of various cytokines that cause fibroblasts to become myofibroblasts [1, 22, 23].

### Previous treatment options of osteoradionecrosis

The management of ORN is difficult and is not always successful because of the lack of suitable, effective methods corresponding to the various lesions of the oral cavity and jaw accompanied by various risk factors. ORN of the jaw is usually treated with conservative or surgical management. Conservative therapies include frequent saline irrigation and antibiotic medications during infectious periods. Another conservative approach is hyperbaric oxygen treatment (HBOT). These treatment options are selected according to the stages of ORN, especially for the effective treatment of early and advanced ORN. Stage I, or early stage ORN, is managed conservatively with therapies such as local wound care, HBOT, and antibiotic medications. Stage III, advanced stage ORN, is managed surgically with wide resection and immediate microvascular reconstruction. For stage II, intermediate stage ORN, it is difficult to recommend a definitive treatment procedure [20, 24–26].

Antibiotic medications should always be instituted after bacterial identification and sensitivity testing, and any delays in surgical treatment should be avoided. Usually, penicillin with metronidazole or clindamycin is initially administered until bacterial identification is available. Previous clinical studies have shown that the polymicrobial nature of ORN presents with a microflora spectrum that is very responsive to the therapeutic regimens normally used to treat odontogenic infections. Consultation with an infectious disease consultant is especially helpful in cases involving long durations of treatment [24, 27, 28].

**Figure 3:**
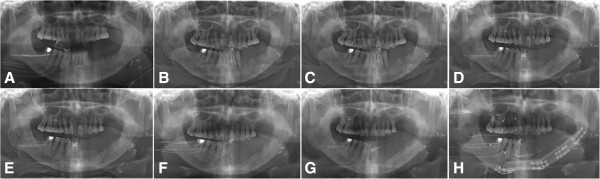
**A serial panoramic views of various surgical procedures in a left tongue cancer patient after glossectomy with radial forearm flap reconstruction (A), with a suspicion for ORN after radiotherapy (B), after wound debridement (C), after sequestrectomy (D, E), after decortication with sequestrectomy (F, G), and reconstruction with a fibular free flap (H).**

Surgical approaches for osteoradionecrotic jaws may encompass a series of procedures, including wound debridement, which involves the removal of infected and devitalized teeth and associated soft tissues, sequestrectomy, which is the removal of devitalized bony fragments or an involucrum of the jaw, decortication, which is the removal of lateral and inferior cortical plates of bone to gain access to the infected medullar cavity, and resection with health bony margins with immediate or delayed reconstruction (Figure 3).

HBOT is well known to positively affect surgical outcomes by promoting angiogenesis in irradiated tissues. HBOT not only increases the oxygen supply in hypoxic tissue, thereby inducing fibroblastic proliferation and capillary formation, but also increases tissue vascularity, viability and healing capacity [29, 30]. HBOT most likely achieves these effects through a complex series of changes in affected tissues. Tissue swelling is probably improved through the osmotic effect of oxygen, and the steep oxygen gradient established across an irradiated tissue margin leads to the growth of new blood vessels. In addition, improving oxygen levels improves white blood cell and fibroblast function, further enhancing wound healing [31]. However, in a randomized control study, HBOT was not better than placebo [30]. In addition, HBOT was associated with many adverse effects including pressure-induced damage to the ears, sinuses and lungs, a temporary worsening of short sightedness (myopia), claustrophobia and oxygen poisoning. Thus, the use of HBOT in treating established ORN has not been convincing and is still controversial.

The indications for an individual or combined approach to treating ORN have not been defined [15, 32]. Recent understanding of the pathophysiology of ORN based on the concept of radiation-induced fibrosis has led to the advent of new therapeutic regimens composed of pentoxifylline and tocopherol [33].

### Oral and maxillofacial complications of radiotherapy

Radiation-induced damage to the oral and maxillofacial region is the result of the deleterious effects of therapeutic radiation on the oral mucosa and the adjacent salivary glands, maxilla, mandible, teeth, and masticatory musculatures. To prevent these complex complications of radiotherapy and to improve quality of life in radiation-exposed patients, cytoprotective drugs, biological response modifiers, improved salivary-sparing radiation techniques, and extensive surgical approaches have been introduced.

The severity of oral complications of radiotherapy ranges from superficial, slowly progressive bone erosion to pathological fracture. Patients often present with signs and symptoms of pain, drainage, fever, and fistula formation. These complications rarely occur in patients who have been exposed to radiation doses less than 60 Gy and are more common when brachytherapy is utilized [1, 21]. Dental and periodontal disease, dental extraction, surgery, and trauma are frequently associated with the onset of ORN [30]. ORN has also been reported to occur spontaneously. There are a number of risk factors that contribute to and are associated with the development of ORN.

To define and grade radiation-induced complications, several classification systems have been developed. The Common Toxicity Criteria of the National Cancer Institute (NCICTC) includes ORN as a musculoskeletal side effect and mainly considers its functional impact. There are also other scoring systems proposed by Store and Boysen, and Glanzmann and Gratz (Table 5) [10, 34]. These systems have not yet been fully validated in clinical practice and modifications are continuing to be proposed.

**Table 5:** **Scoring systems of ORN**

	Score	Event
NCICTC* [31]	0	None
	1	Asymptomatic and detected by imaging only
	2	Symptomatic and interfering with function, but not interfering with activities of daily living
	3	Symptomatic and interfering with activities of daily living
	4	Symptomatic, or disabling
Store & Boysen [9]	0	Mucosal defects only
	1	Radiological evidence of necrotic bone with intact mucosa
	2	Positive radiological findings with denuded bone intra-orally
	3	Clinically exposed radionecrotic bone, verified by imaging techniques, along with skin fistulas and infection
Glanzmann & Gratz [47]	1	Bone exposure without signs of infection and persisting for at least three months
	2	Bone exposure with signs of infection or sequester and without the signs of grade 3 ± 5
	3	Bone necrosis treated with mandibular resection with a satisfactory result
	4	Bone necrosis with persisting problems despite mandibular resection
	5	Death due to osteoradionecrosis

*NCICTC: Common Toxicity Criteria of the National Cancer Institute.

### Pentoxifylline

Pentoxifylline is a tri-substituted methylxanthine derivative chemically designated as 1-(5-oxohexyl)-3,7-dimethylxanthine, and is a hemorrheologic agent, unlike theophylline. Its chemical name is 3,7-dihydro-3,7-dimethyl-1-(5-oxohexyl)-1H-purine-2,6-dione and its molecular formula is C13H18N4O3 with a molecular mass of 278.3. Pentoxifylline is a white to creamy white crystalline powder. It is freely soluble in chloroform and methanol, soluble in water, sparingly soluble in ethanol, sparingly soluble in toluene, and slightly soluble in ether. It has a melting point of 104 to 107 °C, within a 3°C range [22, 23].

Pentoxifylline exerts an anti-tumor necrosis factor (TNF)-α effect, increases erythrocyte flexibility, vasodilates, inhibits inflammatory reactions *in vivo*, inhibits human dermal fibroblast proliferation and extracellular matrix (ECM) production, and increases collagenase activity *in vitro*. Pentoxifylline and its metabolites improve blood flow by decreasing its viscosity. In patients with chronic peripheral arterial disease, this effect increases blood flow to the affected microcirculation and enhances tissue oxygenation. The usual dosage of pentoxifylline in extended-release tablet form is 400 mg, three times a day with meals. While the effect of pentoxifylline may be seen within 2 to 4 weeks, it is recommended that treatment be continued for at least 8 weeks.

The precise mode of action of pentoxifylline and the sequence of events leading to clinical improvement are not yet defined. Pentoxifylline has been shown to increase leukocyte deformability and to inhibit neutrophil adhesion and activation in animal and human *in vitro* studies. Tissue oxygen levels have also been shown to increase significantly with therapeutic doses of pentoxifylline in patients with peripheral arterial disease. Indications for pentoxifylline include the treatment of patients with intermittent claudication due to chronic occlusive arterial disease of the limbs. Although pentoxifylline can improve function and symptoms in the treatment of peripheral vascular disease, it is not intended to replace more definitive therapy, such as surgical bypass or removal of arterial obstructions.

Clinical trials have been conducted using either extended-release pentoxifylline tablets for up to 60 weeks or immediate-release pentoxifylline capsules for up to 24 weeks. Dosage ranges were 400 mg *bid* to *tid* in the tablet studies and 200 to 400 mg *tid* in the capsule studies. The incidence of adverse reactions to pentoxifylline were less than 1%, and general side effects were not typical. Digestive and central nervous system side effects are dose-related. If patients develop these effects, it is recommended that the dosage be lowered to one tablet *bid*, 800 mg/day. If side effects persist at this lower dosage, the administration of pentoxifylline should be discontinued. After its oral administration in aqueous solution, pentoxifylline is almost completely absorbed. It undergoes a first-pass effect and its various metabolites appear in plasma very soon after dosing. Peak plasma levels of the parent compound and its metabolites are reached within one hour. The major metabolites are metabolite I (1-[5-hydroxyhexyl]-3,7-dimethylxanthine) and metabolite V (1-[3-carboxypropyl]-3,7-dimethylxanthine), and plasma levels of these metabolites are 5 and 8 times greater than pentoxifylline, respectively.

Patients on warfarin should undergo more frequent monitoring of prothrombin times, while patients with other risk factors complicated by hemorrhage, e.g., recent surgery and peptic ulceration, should have periodic examinations for bleeding tendencies. In general, dose selection for elderly patients should be cautious, usually starting at the low end of the dosing range, reflecting the greater frequency of decreased hepatic, renal, or cardiac function, and of concomitant disease or other drug therapy. Safety and effectiveness in pediatric patients have not yet been established. Pentoxifylline has been used concurrently with antihypertensive drugs, β blockers, digitalis, diuretics, and antiarrhythmics. Small decreases in blood pressure have been observed, so periodic systemic blood pressure monitoring is recommended for patients receiving concomitant antihypertensive therapy. If needed, dosage of antihypertensive agents should be reduced. Concomitant administration of pentoxifylline and theophylline-containing drugs leads to increased theophylline levels and theophylline toxicity in some individuals. Such patients should be closely monitored for signs of toxicity and have their theophylline dosage adjusted as necessary.

### Tocopherol

Tocopherols are a class of organic chemical compounds consisting of various methylated phenols, many of which have vitamin E activity. Because the compound’s vitamin activity was first identified in 1936 as a dietary fertility factor in rats, it was given the name “tocopherol” from the Greek words “τόκος” (*tókos*, birth) and “φέρϵιν”, (*phérein*, to bear or carry) meaning “to carry a pregnancy,” with the ending “-ol” signifying its status as a chemical alcohol. Tochotrienols are related compounds that also have tocopherol activity. All of these derivatives with vitamin activity may correctly be referred to as “vitamin E”. Tocotrienols have the same methyl structure in its ring and the same Greek letter-methyl-notation, but differ from tocopherols due to the presence of three double bonds in the hydrophobic side chain. Whereas tocopherols have three centers and eight possible stereoisomers per structural formula, the unsaturation of tocotrienol tails has only a single stereoisomeric carbon and, thus, two possible isomers per structural formula, one of which occurs naturally. Vitamin E exists in eight different forms, four tocopherols and four tocotrienols. All feature a chromane ring, with a hydroxyl group that can donate a hydrogen atom to reduce free radicals and a hydrophobic side chain that allows for penetration into biological membranes. Each form has a different biological activity; the unnatural l-isomers of tocotrienols lack almost all vitamin activity, and half of the eight possible isomers of the tocopherols, those with 2S chirality at the ring-tail junction, also lack vitamin activity. Of the stereoisomers which retain activity, increasing methylation, especially full methylation to the alpha-form, increases vitamin activity. Both the tocopherols and tocotrienols occur in α (alpha), β (beta), γ (gamma) and δ (delta) forms, determined by the number and position of methyl groups on the chromanol ring [22, 23, 35].

Tocopherols and tocotrienols are fat-soluble antioxidants, but also seem to have many other functions in the body. The functions of endogenous tocopherol are to scavenge the reactive oxygen species generated during oxidative stress that escape the activity of *in vivo* antioxidant enzymes, to protect cell membranes against lipid peroxidation, and to partly inhibit TGF-ß1 and procollagen gene expression.

### Animal models for the treatment of ORN

Various studies have reported the clinical and radiological features of ORN, but few data are available regarding the histopathologic findings of irradiated bone in human or experimental studies. An animal model would be relevant for evaluating the histopathologic findings of irradiated bone, as well as treatments for ORN with tissue engineering biomaterials [36–41]. In an ORN-induced animal experimental model, osteonecrotic bone cannot be credit significantly after different radiation treatments. The established ORN does not regress spontaneously, and neither stabilizes nor gradually worsens, and so is notoriously difficult to manage [1, 4]. Exposing animals to dental extraction, inoculation of infectious microorganisms, tobacco, and corticosteroids are useful in creating the acute inflammatory reaction associated with radionecrosis.

**Figure 4:**
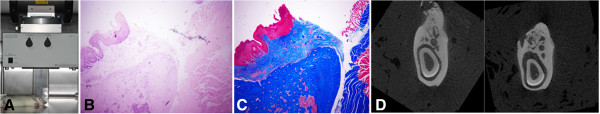
**An experimental osteoradionecrosis rat model, including the apparatus with irradiation (A), and hematoxylin-eosin (B) and toluidine blue (C) staining with micro-computed tomogram views (D).**

The present experimental works were designed to investigate the effects of increasingly high doses of ionizing radiation on the histologic and radiologic findings of the jaw in animal models (Figure 4). All experimental animal ORN models are summarized in Table 6 [42–47].

**Table 6:** **Rat animal models of osteoradionecrosis**

		Radiation irradiation									
Animal	Sample	Source	Fraction dose	Frequency	Interval	Total dose	Irradiation target	Intervention	Time after irradiation	Evaluation	Reference
SD rats	12	Ortho	5.91/7/8.89	5	1d	29.5/35/44.5	Mandible (Left)	Mechanical test	56 days	-	Tchanque-Fossuo et al. (2011) [19]
SD rats male	10	Brachy	20		7d		Mandible (Left)	Extraction	21 days	Radiology, histology	Tamplen et al. (2011) [35]
SD rats	20	Ortho	5.91/7/8.89	5	1d	29.5/35/44.5	Mandible (Left)	Histology only	56 days	Histology	Tchanque-Fossuo et al (2011) [19]
SD rats male	10	Brachy	30	1		30	Mandible (Left)	Extraction	28 days	Radiology, histology	Cohen et al. (2011) [41]
SD rats male	10	Ortho	3.6	10	1d	36	Mandible (Left)	DO unilateral	8 weeks	Histology	Inyang et al. (2010) [43]
Rat		hfSRT	15			60	Mandible (Left)	DO unilateral	6,12 weeks	Radiology , histology	Fenner et al. (2010) [40]
Wistar rats male	108	Co60	8	1		8	Mandible (Left)	Extraction	10,12 days	Histology	Hosokawa et al. (2007) [44]
SD rats male	12	Ortho	3.6	10	1d	36	Mandible (Left)	DO unilateral	8 weeks	Radiology	Fregene et al. (2009) [45]
Rats (WKY, Lewis, Fisher) male	24	Brachy	20	1		20	Mandible (Right)	Injection	7 weeks	Radiology & histology	Springer et al. (2008) [48]
Rats (WKY, Lewis, Fisher) male	24	Brachy	20	1		20	Mandible (Right)	Histology only	100 days	Histology	Niehoff et al. (2008) [37]
Wistar rats	25	6 MV	15	4	2w	60	Mandible (Left)	Histology only	6,12 weeks	Histology	Fenner et al. (2010) [40]
Wistar rats male	30	Ortho	6	7	2 - 3d	42	Mandible (Right)	Histology only	85,141, 253 days	Histology	Williamson R.A (2007) [29]
SD rats male	60	Co60	2.5/3	18/15		45	Mandible (Bilateral)	Bone defect	6, 8 weeks	Histology	Lorente et al. (1992) [46]
SD rats male	10	Brachy	20	1		20	Mandible (Left)	Extraction	28 days	Radiology, histology	Tamplen et al. (2011) [35]
Wistar rats	50	Ortho	20	1		20	Mandible (Left)	Growth only	30, 60 days	Radiology, histology	Ubios et al. (1992) [47]

### Recent reports of pentoxifylline and tocopherol combined therapy

Combined pentoxifylline-tocopherol therapy has been proven effective in reducing chronic progressive septic ORN of the mandible. Because there is currently no standard medical treatment, this approach constitutes a useful alternative to existing therapies in treating ORN. These two drugs act synergistically as potent antifibrotic agents and are available, well tolerated, inexpensive, and beneficial to the patient. Pentoxifylline is a methylxanthine derivative that exerts an negative effect on TNF-α, increases erythrocyte flexibility, dilates blood vessels, inhibits inflammatory reactions *in vivo*, inhibits the proliferation of human dermal fibroblasts and the production of extracellular matrix, and increases collagenase activity *in vitro*. It is given with tocopherol, which reduces fibrosis by scavenging the reactive oxygen species that were generated during oxidative stress, protecting cell membranes against the peroxidation of lipids, and partially inhibiting TGF- β1 and the expression of procollagen genes. In animal studies, neither drug alone was capable of reversing the effects of reactive oxygen species [21]. In addition, pentoxifylline or tocopherol alone were also unable to reverse the development of human fibrosis. However, these drugs were effective synergistically as anti-fibrotic agents.

Despite the few reports in the literature on the management of radiation-induced sequelae, a basic understanding of the mechanisms of radiation-induced fibrosis can be elucidated given its regression after antioxidant treatment with superoxide dismutase [3, 9], as well as with a combination [11]. Combined treatment induced a 66% regression of the surface area of radiation-induced fibrosis after 12 months of treatment in a phase II clinical trial [8]. Similar results were confirmed in an experimental cutaneomuscular radiation-induced fibrosis model—a 70% volume regression after six months of treatments [48], as well as in another randomized clinical trial [38]. Futran et al. [14] showed that 1,200 mg/day of pentoxifylline alone accelerated the healing of mucosal radiation-induced injury in nine of twelve cases of oral soft tissue necrosis without ORN. On the basis of new pathologic understandings of radiation-induced fibrosis, this combination could reverse ORN by reducing the microscopic fibroatrophic changes associated with the progressive necrotic process and by stimulating defective osteoblastic healing [21]. In one woman with an extensive progressive exteriorized ORN of the sternum, complete cutaneous and bone healing was obtained with combined therapy with clodronate, a well-known bisphosphonate that inhibits osteoclastic bone destruction [24, 25]. Between June 1995 and January 2002 in another phase II trial at Saint Louis Hospital in Paris, 18 consecutive patients were treated for severe mandible ORN and chronic persistent ORN with combined therapy, which was boosted with clodronate in the last eight more severe cases of active progressive ORN. All patients showed improvement at 6 months. Sixteen of the 18 patients recovered completely, 14 of whom recovered within 8 months. The remaining two patients responded but not as well [1, 2]. Over 7 years, all of the patients were given daily pentoxifylline 800 mg combined with tocopherol 1,000 IU orally for 6–24 months. The eight more seriously affected patients were also given clodronate, 1,600 mg/day 5 days a week.

Clodronate is a new generation bisphosphonate that inhibits bone resorption by reducing the number and activity of osteoclasts [1, 2]. An ORN-like disease of mandibular bone has been reported in patients given this treatment for cancer-associated hypercalcemia or metastatic osteolytic lesions. Unlike previous bisphosphonates, clodronate has been shown to act directly on osteoblastic cells by increasing the formation of bone and reducing the proliferation of fibroblasts [21].

Continuous treatment of patients with combined pentoxifylline, tocopherol and clodronate has proven effective in reducing chronic progressive septic ORN of the mandible. Because there is currently no standard medical treatment, this conservative treatment approach constitutes a useful alternative to existing therapies in treating ORN. All three drugs are available, well tolerated, inexpensive, and beneficial to the patient. Questions regarding the precise synergistic mechanisms of actions of these drugs will be investigated in a future randomized clinical trial.

## Conclusions

**Figure 5:**
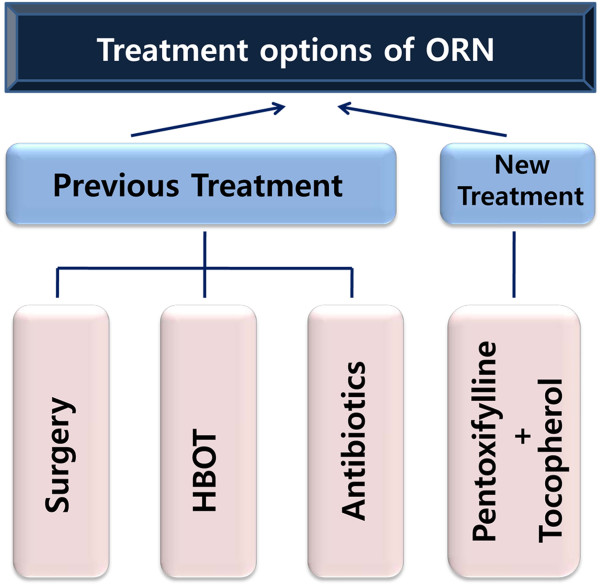
**Recent treatment options for the management of osteoradionecrosis of the jaw.**

Recent advances in the understanding of ORN pathogenesis have resulted in a new therapeutic strategy designed to improve tissue healing with a combination of pentoxifylline and tocopherol. From the published literature on this topic, mainly consisting of retrospective chart reviews, we can conclude the treatment options for ORN of the jaw can be expanded to include combined therapy to accompany the three main approaches; antibiotics, surgery and HBOT (Figure 5). Increasing effects of collagenase activity of pentoxifyllin with fibrosis inhibition effect of tocopherol will be added to these previous treatment options. Histopathological and molecular biological approaches to ORN with radiation-induced fibrosis should be executed in a well-designed animal experimental model before ultimately beginning prospective randomized control trials in the near future.

Histopathological and molecular biological approaches to ORN with radiation-induced fibrosis should be executed in a well-designed animal experimental model before ultimately beginning prospective randomized control trials.

Ethical approval: Not required. - Patient permission: Not required.
